# TC^99m^ MDP bone scan in evaluation of painful scoliosis

**DOI:** 10.4103/0972-3919.72691

**Published:** 2010

**Authors:** Sujit Nilegaonkar, Sameer Sonar, Ashish Ranade, Madhav Khadilkar

**Affiliations:** Shreemati Kashibai Navale Medical College & Hospital, Narhe, Maharashtra, India; 1 Ruby Hall Clinic, Pune, Maharashtra, India

**Keywords:** Bone scan, osteoid osteoma, painful scoliosis

## Abstract

A 18-year-old male presented with low back ache. The patient was investigated and was diagnosed to have painful scoliosis. X-ray and other examinations could not reveal any diagnosis. The patient was referred to undergo bone scan on clinical suspicion of osteoid osteoma and to rule out stress fracture if any. Planar bone scan was performed, which showed a lesion in L3 vertebra and was further evaluated with SPECT (Single photon emission computed tomography) study to characterize the lesion. On SPECT examination, the classical features of osteoid osteoma, the double density sign (11), was noted in the pars interarticularis region. These findings were confirmed by a CT scan, which showed a sclerotic lesion in pars interarticularis of L3 vertebra. The patient was posted for operation and was relieved of symptoms in the postoperative follow-up.

## INTRODUCTION

Osteoid osteoma is one of the most common benign tumors of the spine and it is also the most common cause of painful scoliosis in adolescents.[1–3] Up to 20% of osteoid osteomas are found in the spine, of which 60% are located in the lumbar spine and 27% in the cervical spine, 12% in the thoracic spine, and 2% in the sacrum. Osteoid osteoma of the spine must be considered when back ache is associated with muscle spasm and scoliosis. Radiographs often miss the diagnosis due to their small size and the complex anatomy of the spine.

Two thirds of spinal osteoid osteomas represent as painful scoliosis. It is, however, very common to miss the diagnosis and the patient may be subjected to treatments, such as bracing, adding discomfort to the patient.[4]

## CASE REPORT

An 18-year-old male presented with low back ache since 1 year. The patient had scoliosis. Typical evening raising plain X-ray was done and was inconclusive. Bone scan was ordered to evaluate boney pain.

A three-phase bone scan was performed. The planar images revealed a focal solitary lesion in the L3 vertebra. The lesion was further evaluated with SPECT study for further characterization, which revealed a lesion in the L3 vertebral pars interarticularis region. Central focal hot spot with peripheral decremental uptake was noted in L3 vertebral pars interarticularis.

**Figure 1:**
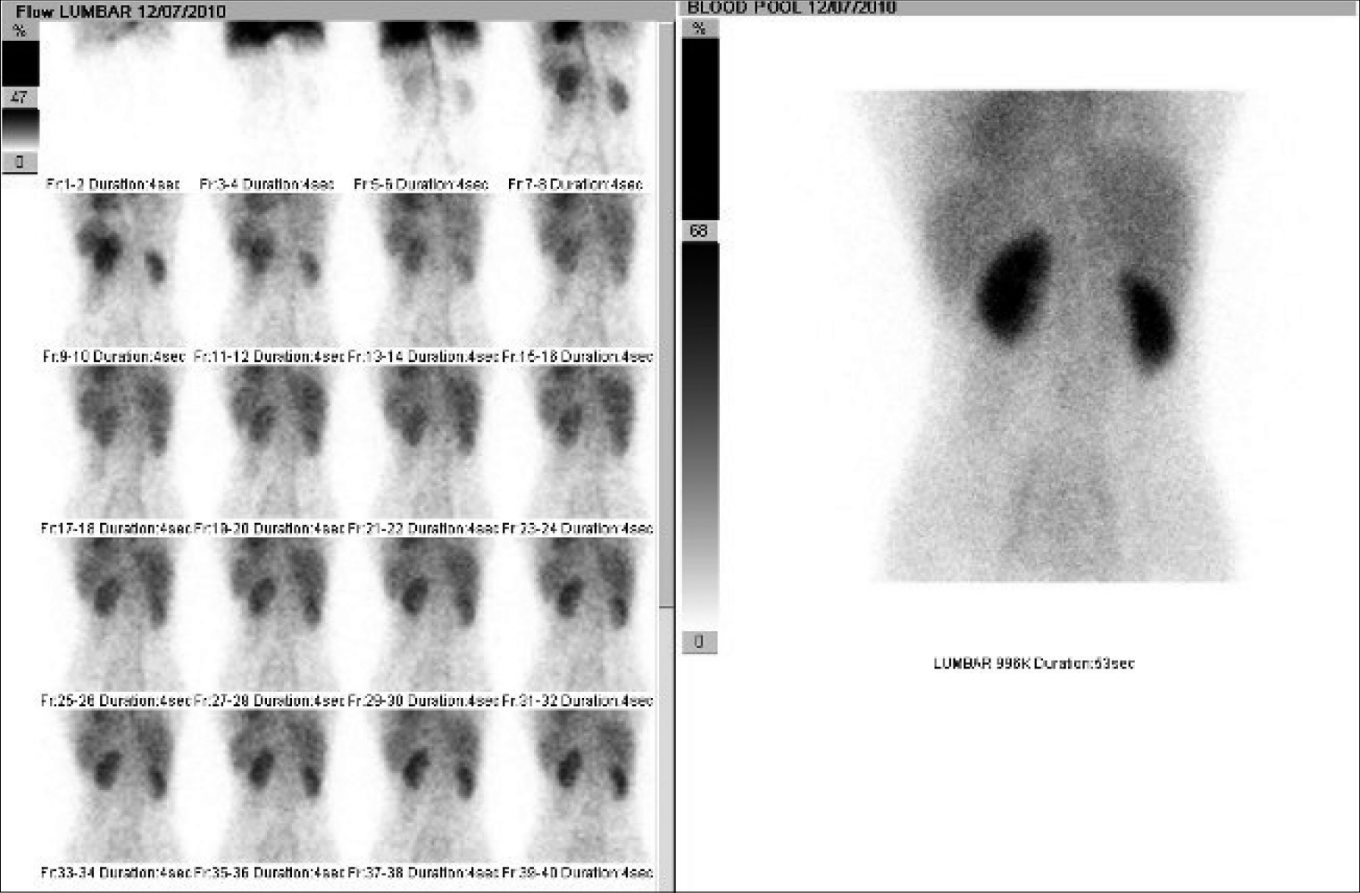
Flow and blood pool images showing normal perfusion and blood pooling of the tracer in the lumbar region

**Figure 2:**
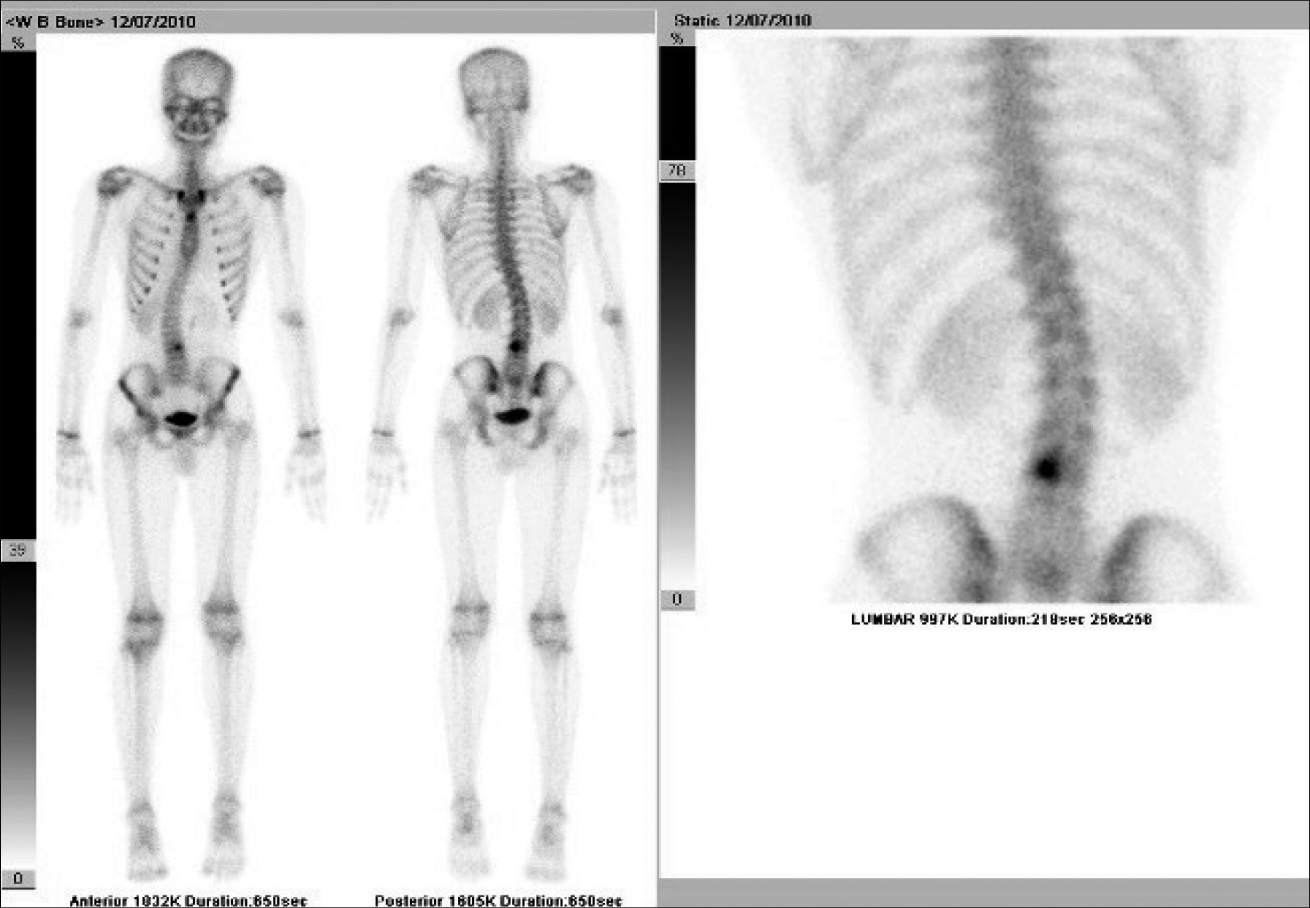
Whole body bone scan performed with 20 mCi of Tc^99m^ MDP (methylenediphosphonic acid): showing focally increased tracer uptake in the L3 vertebra on the left side. Note lumbar scoliosis with convexity towards right side

**Figure 3:**
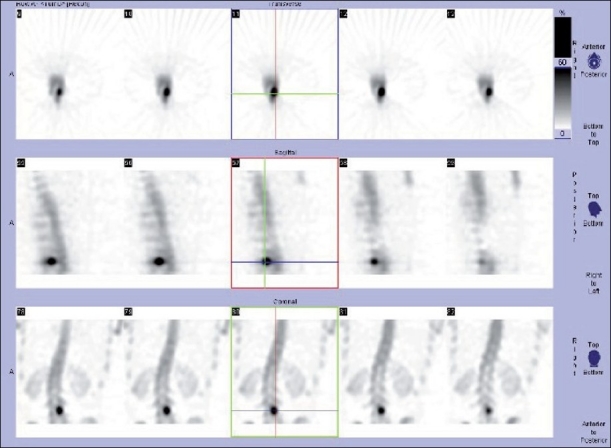
Further evaluation of the lesion with SPECT study shows central intense uptake with peripheral decremental tracer uptake (double density sign) noted in the left pars interarticularis of L3 vertebra

**Figure 4:**
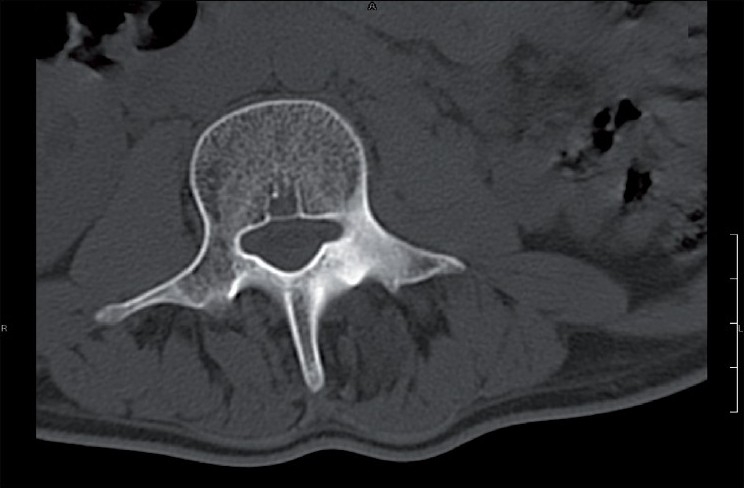
Sclerotic lesion in pars interarticularis of L3 vertebra

A CT scan was performed to precisely localize the lesion and to confirm the diagnosis. CT scan showed osteosclerotic lesion in the L3 vertebral pars interarticularis region [Figure 1–4].

## DISCUSSION

Osteoid osteoma is a benign osteoblastic neoplasm most often seen in young males, frequently in the first 3 decades of life and may be found in the cortical or cancellous bone with a further 5% reported as subperiosteal, and multicentric foci were also reported.[5,6]

Preliminary diagnosis carries some difficulties, mostly due to unawareness of the condition, leading to delay in the management. The first imaging procedure is usually the plain radiography,[4,6–8] which shows different pictures depending on the location of the lesion (whether medullary or cortical) and the degree of reactive sclerosis surrounding the nidus. The lesions in the pelvis and spine are usually difficult to identify by plain radiographs.[4,6,7,9] The most important plain radiographic differential diagnosis includes osteoblastoma, Brodies abscess, stress fracture, and enostosis.[1,4]

Although radioisotope imaging is not pathognomonic for osteoid osteoma, it can be very useful in diagnosis and anatomic location, and intraoperative and postoperative identification.[3–5,7–10] The sign of “double density image” is usually created,[11] which is fairly typical of osteoid osteoma.[5,7]

A CT scan is essential for optimal planning before surgery is performed and for postoperative confirmation of complete resection.

Treatment can be surgical or conservative. To achieve a surgical cure the entire nidus must be removed completely; medical management of osteoid osteoma is well recognized and is usually needed whenever the lesion is inaccessible even with the most carefully planned surgical exposures, or is adjacent to vital neurovascular structures, or when the patient refuses any operative interference.

In conclusion, any child or adolescent with scoliosis complaining of back pain, particularly long-standing, should be investigated thoroughly, keeping in mind the possibility of osteoid osteoma and a bone scan should be ordered as a primary investigation because a plain radiograph may not be significant in detecting these lesions.
